# The Institutional Positioning of Environmental Tort Remedy in China: Executive-Led or Judicial-Led?

**DOI:** 10.3390/ijerph20021443

**Published:** 2023-01-12

**Authors:** Tian Sang, Lijun Zhang

**Affiliations:** Koguan Law School, Shanghai Jiaotong University, Shanghai 200030, China

**Keywords:** environmental tort, public interest litigation, environmental administrative power, judicial remedy, risk communication

## Abstract

There are two options for environmental tort remedy in China: resorting to environmental administration or environmental justice, with an ongoing debate over which of the two should lead. Firstly, it compares the structure of China’s environmental tort remedy system and the two types of power: administrative power and judicial power, concluding that administrative power is dominant. Then, it argues for the indispensability of judicial power, attempts to find a clear boundary between the two sides, and justifies their mutual division of labor and collaboration. Through sufficient demonstration, it clarifies why the dominant position of environmental administrative power must be guaranteed. Then, it summarizes the experience of other countries and the practice of environmental protection in China; and provides three innovative paths of the future environmental rights remedy system. These three aspects are setting up a review procedure for administrative priority judgment before filing an environmental lawsuit, establishing the independent position of experts in environmental litigation, advocating a risk communication mechanism other than litigation, and providing a richer institutional guarantee for the relief of environmental rights.

## 1. Introduction: What Is the Leading Power in Environmental Tort Remedy?

After the introduction of the Civil Code of the People’s Republic of China, the environmental code is considered to be the most promising department law to codify next [1]. The legislative work plan for the year 2021 has clarified this, and China has proposed that it will soon investigate and initiate codification work in such areas as environmental code, education code, and basic administrative code [2]. Before that, China had built a comprehensive environmental management system since the 1980s, with legislation and administrative enforcement as the primary means and judicial litigation as a secondary means.

China’s extensive legal systems for environmental protection are comprised of a series of legislative and administrative regulations. The Environmental Protection Law of the People’s Republic of China (2014 Revision) defines the purpose of China’s environmental legislation to protect the environment and facilitate sustainable economic and social development [3]. In line with this dominant law and this basic legislative purpose, China has set up preventive environmental civil public interest litigation in the last decade and has accumulated a wealth of cases and experiences so far. The most notable sign in this regard is that China expanded the eligibility of litigation subjects when it amended the Civil Procedure Law in 2012, expanding the eligibility of plaintiffs and the scope of public interest litigation in environmental civil cases. It is also retained in the latest revision of the Civil Procedure Law in 2022, with Article 58 stipulating that “Legally designated institutions and relevant organizations may initiate proceedings at the people’s court against acts jeopardizing public interest such as causing pollution to the environment or damaging the legitimate rights or interests of consumers at large [4].” In this way, China’s judicial protection of the environment is safeguarded by both substantive and procedural laws.

However, can judicial power, especially civil environmental public interest litigation, become the dominant force in environmental tort remedies? Given China’s extensive body of laws and regulations, is judicial power the main option for citizens or social organizations to resort to when the environment is harmed? The answer is still no, and this role is still played by the administrative bodies represented by government departments. This paper intends to make a detailed demonstration of the main logic and historical changes of this core argument. It particularly emphasizes that such a pattern of “administrative power dominance” is not due to the expansion of public power, but by the realities of China. In this paper, a comparative approach will be introduced, and the practical experiences of other countries will be reviewed for comparison.

## 2. Framework of the Environmental Tort Remedy System in China

Under the premise of legal rights, a system is in place to remedy and compensate for violations of rights when they occur. If the protection of rights before they are violated is a confirmation of rights, the protection of rights after they are violated is a remedy of rights, as defined in this paper. The protection of both types of rights requires a legal basis. In China, in addition to the Environmental Protection Law and the Civil Procedure Law mentioned above, there are also a series of judicial interpretations and more detailed lower-level laws that further specify the rights related to environmental law. Representative separate laws include the Marine Environmental Protection Law (2017), the Atmospheric Pollution Prevention and Control Law (2018), and the Water Pollution Prevention and Control Law (2017).

Environmental law is clearly distinguished from general private law, and its public interest nature determines the unique design of both rights holders and litigation procedures. For example, many countries, including China, have established the system of civil public interest litigation from the legislative process. In other words, they remove the conditions for filing lawsuits that “the plaintiff has a direct interest in the case” and expand the scope of the court’s jurisdiction. It can also bring environmental tort lawsuits against subjects who are not directly interested. In 2015, China promulgated the Interpretation of the Supreme People’s Court on Several Issues concerning the Application of Law in the Conduct of Environmental Civil Public Interest Litigations. It emphasizes the preventive and public interest aspects of environmental tort remedy and tries to stop the infringement before the consequences become generalized. Therefore, the plaintiff can bring a lawsuit only if the following conditions are met: “a preliminary proof that the defendant’s actions have a material risk of damaging the public interest of the society [5].”

Based on the framework built by these basic laws, China has developed a system of laws, regulations, and policies on environmental rights protection after a long-term empirical exploration. These include the Interim Measures for Hearing the Administrative License in Respect of Environmental Protection in 2004, the Measures for Public Participation in Environmental Protection in 2015, and the Measures for Public Participation in Environmental Impact Assessment in 2018. These regulations establish the basic principles and basic ways of public participation in environmental protection, and these ways of defending the rights are linked to the public power operation of the administration and justice of environmental protection. The Kunming Declaration of the World Judicial Conference on Environment of May 2021 explicitly advocates the adoption of active preventive judicial measures to prevent the occurrence and expansion of ecological damage, which also explicitly provides concrete measures such as injunction and security pre-litigation [6].

On the surface, environmental judicial trials are the dominant force in China’s current efforts to defend environmental rights under two premises. First, there is a complete legal system for environmental protection. Second, China has set up a preventive public interest litigation system for civil environmental cases. However, in the current practice of environmental tort remedy in China, from 2015 to 2019, there were only 5184 cases of environmental public interest litigation in China [7]. There are only 3619 cases in the year 2020 for which the most recent data are available [8], but as many as 833,000 cases were handled by administrative means such as administrative penalties [9]. This disproportionate data seems to indicate that the vast majority of environmental tort cases are ultimately resolved through the operation of administrative power. Indeed, administrative enforcement of environmental cases is superior to judicial enforcement, both in terms of professionalism and efficiency, and it is consistent with China’s institutional tradition of administrative superiority. Therefore, the question of whether environmental cases should be handled in an executive-led or judicial-led manner has become a practical issue in China and is the main concern of this paper. We can compare these two powers in the following three aspects.

### 2.1. Functional Orientation of Public Power and Equity

First of all, we should clarify that in the field of environmental rights remedy, administrative power and judicial power work in cooperation with a due division of labor. Tort liability is originally designed to cover damages. If damages have not yet been incurred or have not amounted to the extent that they need to be remedied, then the judicial activity would contradict the principle of judicial neutrality and passivity. In contrast, in this scenario, administrative power has the distinct advantage of being efficient and proactive. The most important administrative initiative is to directly exercise the picketing power to make impact-oriented interventions against parties causing large-scale environmental damage. At an earlier stage, government agencies can make proactive assessments even before environmental risks are posed. For example, China has adopted official regulations that define the procedures for significant administrative decisions, specifically stating that “actors shall assess the controllability of risks for decisions that may adversely affect the ecology and environment [10].” On the other hand, we can also consider it from the perspective of fairness. Judicial power is naturally neutral and negative, serving as a neutral judge for both parties in the dispute. If such a judge is personally involved in the game, their work balancing interests can hardly be considered fair. Moreover, even if there is an obvious bias in the decision of the case, it can hardly be corrected by using judicial power. Therefore, the overdraft of a second remedy may lead to an inability to find a suitable way to relieve the remedy, as evidenced by the experience of legal exploration in various countries.

### 2.2. Expertise and Skills

The expertise of judicial power is in interpreting and applying legal provisions and regulations, which corresponds to rules. In contrast, the expertise of administrative power is in responding to and resolving complex and specific situations, which corresponds to reality. From the perspective of balancing of interests, administrative agencies are often positioned as representatives of public interests. Especially in the field of environmental protection, the basic task of environmental law enforcement is to protect the public interest and implement public policies. The more unclear the subject of interest is, the more prominent this representative effect of the administrative agency becomes. In contrast, the judicial power or the judiciary apparently lacks the technical and justified status of such representation. Especially in recent decades, the concept of integrated ecosystem management (IEM) has been gradually accepted as a systematic approach for the integrated management of natural resources and the natural environment. It requires an integrated approach to the components of the ecosystem, considering social, economic, and natural needs and values, and applying multidisciplinary knowledge and methods [11]. Only administrative power is compatible with this concept. In China, for example, the Ministry of Ecology and Environment is the environmental administration agency under the State Council, and it has developed an administrative system with a full range of responsibilities for enforcement, supervision, and management. Each of these agencies has a clear division of responsibilities. Beyond the Ministry of Ecology and Environment, the Ministry of Natural Resources, also an administrative agency, assumes the corresponding supervisory and management responsibilities [12]. It is equivalent to the division of the administrative system into more complex subsystems, each of which has expertise, skills, and a reserve of experience to deal efficiently with various situations.

### 2.3. Effectiveness of the Implementation of Environmental Protection Objectives

The above analysis is mainly conducted to compare administrative and judicial power. However, if we go back to the question, administrative enforcement has distinct advantages in terms of the effectiveness of environmental and ecological protection. A typical example in the professional field of environmental protection is ecological and environmental monitoring and evaluation. The courts are not competent in tracing the source of pollution or even extracting and testing chemical pollutants. Even if a plaintiff initiates a preventive lawsuit, it often needs to connect with the environmental administration enforcement department for intervention. Judicial remedy for environmental protection is a necessary complement to environmental administration enforcement, which is a relatively uniform institutional orientation in the environmental protection legal systems of countries today. In the case of Tennessee Valley Authority v. Hill, known as the first environmental case in the United States, the main objective of the claim was to urge the administrative agency to enforce the Endangered Species Act of 1973 [13]. Since this case is so well known and representative, we do not need to elaborate too much on its details. However, if we extract its main legal relations, we can conclude that the plaintiffs did not want to defend their already violated interests but protect the endangered species from permanent extinction [14]. The lawsuit is ostensibly a request to stop the construction of the dam, but in essence, it is a request for the environmental administration to fulfill its enforcement obligations: a typical administrative lawsuit. The conflict between the snail darter fishes and the dam is still really a balance of interest between individuals. For the citizens and environmental organizations that initiated the public interest litigation, whether the opponent was the dam construction company or the Tennessee Valley Authority, their objective in initiating judicial remedy was to bring the rules of environmental protection law into play, which was evidenced by the argumentative tactics of both sides of the defense in this case [15].

In conclusion, environmental administration remedy is generally prioritized in terms of the essential objectives of environmental protection and the practical effects of its implementation. Dispute handling and resolution through administrative agencies is often considered the most direct, effective and cost-efficient way. Compared with the above adjectives, the judicial route is more indirect, less efficient, with more time, energy and financial resources consumed. There is a traditional Chinese fable called “Yue Zu Dai Pao”, which means a stranger without cooking ability will oversee the cooking work. It is obviously wrong to replace the chef with someone without professional skills, which cannot lead to delicious food and even worse. In the context of functional differentiation in modern society, administrative authorities tend to have more definite advantages in terms of knowledge, finance and experience. In particular, the initiative and professionalism of administrative power in prevention and emergency response are superior to those of judicial power. However, here, we also have a question: Since the advantages of remedy from administrative power are so apparent, why do we need to consider judicial remedy?

## 3. Reasons for Continued Need of Judicial Remedy

According to the above statistics, it can be seen that the number of judicial cases is almost negligible. It seems that the more cases are resolved in the administrative stage, the fewer cases are brought to the judicial channel, and there seems to be a competitive trade-off between the two. Superficially, the legitimacy of the judicial remedy route itself has been questioned. However, as we will demonstrate in this section, the two are still complementary. Preventive public interest litigation for civil environmental cases is an external remedial mechanism that serves the function of interest remedy in the event of a failure or substantial error in environmental administration. From this perspective, remedy from judicial power is also indispensable.

There is a clear logical clue behind the remedy of environmental tort:

(A) The environmental public interest is damaged, but the administrative enforcement of environmental protection fails to work;
(B) Through corrections by administrative agencies, environmental protection infringement has been compensated and remedied.

The other corresponding logical clue is:

(a) The environmental public interest is damaged, but the administrative enforcement of environmental protection fails to work;
(b) The failure of environmental administration enforcement renders the reality of damage to environmental public interest unchangeable or even worse;
(c) The legislation grants the proper subject to initiate public interest litigation, and such subjects may be citizens and environmental organizations whose interests are not directly infringed upon and who can urge environmental administration to enforce the law.
(d) The remedy of the interests, which should have been protected at the first level, is achieved through the second level of judicial remedy.

So, we can find out the role played by the judiciary. The first level of the chain from A→B, which should have been completed, is complemented by the second level of a→b→c→d. In terms of results, the effects of “B” and “d” are equivalent, and they both belong to the social effects achieved after the operation of public power.

The above logical clue is placed in the Environmental Protection Law of the People’s Republic of China, as amended in 2014, which clearly states the principle of “strengthening environmental protection mainly by administrative power rather than judicial power.” Based on the above-mentioned clue, it can be seen that environmental public interest litigation has never undermined and usurped the administrative power, and it still locates itself as a complement to environmental administration enforcement. Historically, such principle of the power for initial administrative judgment originated from the Japanese administrative law theory. It emphasizes that when both the administrative authorities and the court have jurisdiction over a case, the administrative authorities shall have the power to make the initial judgment and deal with the case; the court can generally only proceed with the trial after the administrative authorities have made the initial judgment. The Code of Administrative Court Procedure (Verwaltungsgerichtsordnung) also contains relevant provisions, and its Article 113(5) stipulates that “Insofar as the rejection or omission of the administrative act is unlawful and the plaintiff’s rights are violated thereby, the court shall announce the obligation incumbent on the administrative authority to affect the requested official act if the case is mature for adjudication. ……” [16]. This respect for the power for initial administrative judgment is based on the principle of separation of powers, the characteristics of judicial power under the state system of justice, and the advantages of administrative power, which is fully consistent with the preceding part of this article.

The Supreme People’s Court of the People’s Republic of China also explicitly stated that it “respects the power for initial administrative judgment” in the Ten Cases regarding Government Information Disclosure by the Supreme People’s Court in 2013, which was published in September 2014 [17]. It corresponds specifically to the case of Peng Zhilin v. Changsha County Bureau of Land and Resources of Hunan Province [18]. The Supreme People’s Court noted that the case was typical: “The court considered that whether the government information in question should be provided was subject to the defendant’s investigation and discretion. Therefore, the court’s decision to re-respond is also a respect for the power for initial administrative judgment [19].” The original purpose of establishing this principle is to respect the efficiency and professionalism of the administrative power but also to prevent the inaction of government departments through the supervision of the judicial power so as to regulate and stimulate the administrative power to perform its duties properly. The term “court” here generally refers to a court of first instance, especially a grassroots court.

Why is the judicial remedy system essential? The most important reason is that modern society is functionally differentiated, and the legal system is designed to meet society’s normative and stable expectations, which requires the consideration of the fairness of interests and peace of order. In this context, judicial power has a preset device of a corrective mechanism against administrative power. It allows for a remedy for the wrong results of the administrative power, and this remedy can in turn sustain the administrative power, preventing such a more proactive power from being questioned and thoroughly denied due to misjudged cases. Therefore, we cannot let judicial power replace administrative power nor can we just let administrative power dominate and discard the way of judicial remedy. If judicial power performs the role that administrative power is supposed to play, no one plays the role of judicial power itself, and after all, the solution to deviation in judicial power is often to resort to judicial power of a higher authority. So, we have grounds for believing that the corrective effect of judicial power is equivalent to a safety valve, which is indeed a restriction, but that restriction is also a shield. It is neither a substitute nor a veto. Another special consideration here is the issue of universality of validity. Throughout the world, the abstract administrative act by administrative agencies, which is generally binding, is more conducive to environmental protection in terms of effectiveness. If administrative power is intended to cover as wide a range of circumstances as possible, judicial adjudication is more oriented toward case-by-case remedy, especially given the very specific and unique circumstances of individual cases.

In practice, the coordinated operation of such judicial power in China also has historical roots. Comparable to the case of Tennessee Valley Authority v. Hill for the protection of fish populations in the United States, a typical case for the protection of green peafowl was brought in the southwestern province of Yunnan in China [20]. In this trial, the court ruled that the defendant was required to complete an environmental impact assessment [21]. This assessment should be performed in accordance with the standards of the Ministry of Ecology and Environment, and it is up to the environmental administration to accept and file whether the testing and maintenance measures have been put in place. Ultimately, the administrative authorities provide professional advice on whether to build a hydropower plant that would affect the survival of peacocks. The court simply decides between “accepting” and “not accepting”, and if it refuses to accept, it needs to provide more professional and rigorous detailed arguments, which increases the demand for expertise. Courts that take a negative position often choose to respect and accept the executive branch’s preference.

We can also respond to the questions through the preventive litigation system. The special public interest of environmental protection litigation requires a more complex institutional design than criminal and civil cases, and “prevention beforehand” becomes a necessary complementary dimension to “remedy afterwards.” For this reason, preventive litigation was initiated in China. In other words, before the infringement acts that harm the public interest of the environment are amplified and cause serious consequences, we can interrupt such harm by means of power and make active interventions. China’s Environmental Protection Law clearly states the principle of “according priority to protection, emphasis on prevention” in the General Provisions (Article 5). It also provides for an environmental monitoring system (Article 17) and a “Three Simultaneities” system (Article 41) in the form of explicit legislative provisions. These institutional configurations are evidently implemented based on respect for administrative power, such as the “Three Simultaneities” system, which is an innovation of China in environmental protection. The Environmental Protection Law, which came into force in China in 2005, explicitly stipulates that “Installations for the prevention and control of pollution at a construction project must be designed, built and commissioned together with the principal part of the project. Installations for the prevention and control of pollution shall be examined and considered up to the standard by the competent department of environmental protection administration that examined and approved the environmental impact statement. Installations for the prevention and control of pollution shall not be dismantled or left idle without authorization [22].” The so-called simultaneous design, construction, and commissioning is a typical consideration for preventing environmental tort. Although it is a legislative approach, it supports the operations of both judicial power and administrative power.

Environmental protection in China is clearly divided into two phases. In the early stage, China emphasized economic construction alone, and environmental protection awareness was a relatively minor issue. Any environmental lawsuits that do arise tend to involve clear and simple cases that are primarily aimed at building environmental awareness. At this stage, the typical cases established by the judicial power were instructive and carried the task of educating the general public and business owners to develop an awareness of public environmental protection. However, in the recent stage, there are fewer and fewer such inspiring and abstract representative cases, and the judicial trials of environmental protection are more compatible with the more complicated, standardized and scientific environmental governance. It corresponds to a more economically developed and socially progressive stage in China. In this context, judicial power has made more concessions to administrative power. It is not caused by the proliferation of administrative power but rather by the logical need for a more complex stage of environmental governance. At the same time, judicial remedy can reinforce the implementation of the principle of prevention in environmental law. That is, the precedent constraints allow subsequent cases of similar details to be resolved, and to be dealt with in advance, even before greater harm has occurred. The legal remedy is authoritative not because it is harsh but because it is necessary or inevitable. The certainty of the outcome of the remedy is “predetermined and inevitable.” Given this, administrative agencies and the party responsible for the infringement are often guided in their acts before the remedy of infringement.

## 4. Possible Evolution of China’s Environmental Protection System in the Future

Based on the above discussion, we can forecast an outlook on the system of environmental tort remedy in this section, considering the judicial practice in China and the institutional design of other countries. We suggest that it might evolve in the following three directions in the future:

### 4.1. Setting up a Pre-Litigation Confirmation Process for Administrative Power Review

First, a filter may be set for environmental litigation grounded in respect for the power of initial administrative judgment. In other words, judicial power should adhere to the negative position as a complement to administrative power. Moreover, before initiating a lawsuit to defend the public environmental interest, the parties concerned should have already initiated environmental administration enforcement and exhausted the possibilities of solving the problem through administrative power alone. In environmental litigation involving administrative enforcement, a pre-requisite review procedure for filing a lawsuit should be set up, and a lawsuit that fails to meet this review condition should not be filed.

According to overseas experience, for example, in the United States, whether the plaintiff files a lawsuit against a polluter or an administrative agency, it shall notify the corresponding administrative agency before the lawsuit is initiated. In addition, the plaintiff is not allowed to file a lawsuit within 60 days from the date of notification (except in the case of extraordinary circumstances, such as an emergency where a toxic pollutant is spreading). If the relevant administrative agency with a duty to act is already enforcing the law or has already taken the lead in seeking judicial remedy against the polluter, other citizens and environmental organizations cannot bring a lawsuit [23]. A typical example here is ENGO. ENGO is a non-profit private organization that is dedicated to environmental protection, has no administrative power, and provides environmental public interest services to the community. ENGOs are environmental organizations that are voluntarily initiated by citizens and founded on a common belief. Such organizations do not have public power and are positioned to urge the government to improve or enforce environmental laws and regulations. Historically, these public interest organizations have only brought citizen suits against administrative agencies for their inaction, and monitoring the active enforcement of administrative agencies is an essential element of the lawsuit. For example, wildlife conservation organizations have brought lawsuits against the U.S. Federal Environmental Protection Agency to demand that the government fulfill its environmental protection responsibilities through the interpretation and application of the National Environmental Policy Act (NEPA) and the Endangered Species Act (ESA) [24]. In terms of procedures, the ENGO-initiated public interest litigation in the U.S. against the inaction of administrative agencies is representative, and the procedural provision of prior notice can prevent administrative agencies from failing to fulfill their environmental enforcement obligations and effectively prevent the abuse of environmental public interest litigation.

In Germany, if an environmental public interest organization intends to file an “altruistic environmental group lawsuit” [25], it should first request the administrative authorities to perform their functions. Only if the administrative agency fails to take measures within three months can the organization request the court to make a decree, such as a judgment that the administrative agency orders the operator to take preventive measures, forming a structure in which A urges B and B orders C to act. Whether the judicial remedy for environmental tort is positioned as a civil public interest litigation or an environmental administration lawsuit, the prevailing institutional design requires that the plaintiff first seek to resolve the problem by means of environmental administration before filing a lawsuit. Even when judicial proceedings are initiated, they often involve the judiciary urging the administrative agencies to exercise their powers. In short, the administrative agencies are ultimately responsible for proactive action.

When shifting our focus to the Asian region, we can find that the developing countries are similarly considering the position of judicial power. In the Case of M.C. Mehta v. Union of India, the Supreme Court of India upheld the right of the residents of Delhi to act as plaintiffs in the public interest, holding that plaintiffs have the right to use the courts to require administrative agencies to fulfill their obligations under the law to guarantee the execution of laws and policies [26]. Here, the judicial power also takes a proactive approach but urges administrative power to fulfill its responsibilities separated by a sound wall from the practical initiatives of effective environmental administration. Except in cases of extreme urgency, courts may adopt interim orders. For example, the court may simply order the plant to be shut down in an emergency where a gas leak is ongoing. In such cases, some discretion should be given to the court. After all, the court is endowed with public power to give orders. The case in question is M.C. Mehta v. State of Shriram Food and Fertilizer Industries [27]). In Taiwan, China, there is a similar provision in Article 23 of the Environmental Impact Assessment Act. It stipulates that “When the developer violates this Act or related orders determined pursuant to the authorization of this Act and the competent authority is negligent in implementation, victims or public interest groups may notify the competent authority in writing of the details of the negligent implementation. For those competent authorities that have still failed to carry out implementation in accordance with the law within sixty days after receipt of the written notification, the victims or public interest groups may name the competent authority at issue as a defendant and directly file a lawsuit with an administrative court based on the negligent behavior of the competent authority in fulfilling its implementation duties in order to seek a ruling ordering the competent authority to carry out implementation [28].”

What is more complicated here is that environmental law issues are elevated to the constitutional issues, while it is relatively controversial among countries. Public interest litigation in India can be brought to the level of constitutional litigation, which allows for a judicial review of abstract administrative acts and even legislative acts, with a focus on governmental violations. In the case of Maneka Gandhi v Union of India on 25 January, 1978, the Supreme Court of India stated that the courts can subject any legislation to strict judicial review, even based on the content of the due process clause [29]. This case has been described as a landmark case in Indian judicial history, as the Court significantly expanded the interpretation of Article 21 of the Constitution of India. It overruled A. K. Gopalan v. State of Madras, which had implied the exclusiveness of fundamental rights [30]. However, such cases are historically rare, probably related to the political situation of a country at a specific stage, and they are hardly involved in the routine administrative and judicial areas of environmental law [31].

To sum up, it is a result of delimitation and harmonization to position environmental litigation as an auxiliary or even complementary process to environmental administration enforcement. In the light of the experience of other countries and the results of Chinese practice in environmental protection, it is necessary to set up a review process before an environmental case is filed.

### 4.2. Introducing Experts or Institutions with Expertise as Participants in Litigation

Environmental public interest litigation settings vary from country to country, but they are often characterized by public law litigation behind civil tort cases [32]. Since public interest is involved, we can also look beyond the “judicial/administrative” dichotomy to a broader field. Just as environmental organizations may initiate public interest litigation, private experts and organizations may also accumulate a wealth of expertise in the field of environmental protection as society has evolved to a certain point. While coordination by the environmental administration’s enforcement division is needed, the courts may recruit a wider range of talents as environmental public interest litigation cases grow. They are independent of the operation of public power but can constitute a pool of experts to assist in the trial. In China, the current exploration in this area is mainly concerned with the expert assistant system, the people’s assessor system, and the expert advisory committee system [33]. More developed countries in Europe and the United States also have made many explorations and accumulated sufficient experience in this area, and China may draw reference from the institutional design of other department laws, such as the system of appraisers and the configuration of technical investigators in intellectual property rights.

In environmental public interest litigation, since the applicant may be an individual or a group with no direct interest in the case, the applicant is likely to be unfamiliar with the facts of the case. In such cases, the judge hearing the case needs detailed and comprehensive written testimony from the relevant government department. In India, for example, there are currently Commissions of Implementation to investigate the implementation of environmental administration. However, the effectiveness of such investigations is highly dependent on expert knowledge. In this context, experts’ groups can be introduced and given appropriate status in the legislation. China originally designed the environmental risk control system to prevent infringement by bringing into play the knowledge and technology of experts under the leadership of the administrative power. Environmental public interest litigation deals with the damage to the environment but not the damage to human beings. In areas where public interest arises, expert knowledge represents the level of society, not just the interests of one or two particular individuals. Although this is closely related to the fundamental interests of human beings, it cannot be reduced and divided to specific personal interests. In the design of this approach, experts and the public are two distinct groups [34].

As a specialized litigation, environmental public interest litigation requires not only the presence of people with specialized knowledge but also a clearer and more appropriate status for them from the level of rights remedy, such as the establishment of an expert assistant system [35]. As a result, the litigation rights of the interested parties can be protected to the maximum extent. By using a special group of experts with multiple roles as the hub, we can coordinate the judicial and administrative authorities and ease the burden of proof for environmental litigation applicants. For example, experts can participate in environmental public interest litigation as appraisers, agents ad litem, witnesses, and supporters, and their opinions have evidentiary effect. Thus, some scholars have suggested that the whole group of “experts” can be characterized as a new category of independent litigation participants who can participate in litigation in the name of expert assistants, integrating the above multiple roles. Alternatively, it is suggested that the scope of cases in which the right to sue can be exercised should at least be expanded to incorporate social groups and those who are not directly interested [31].

In this way, we can identify the interaction mechanism between two types of subjects: the “public power subjects” which are mainly courts, prosecutors, and environmental administrations, and the “private power subjects” which are mainly environmental public interest organizations and experts with relevant knowledge. The voice of society has been expressed, and it is the cooperation of social forces and public knowledge. This practice of interaction between multiple subjects within the environmental tort system enables the formation of two complementary branches with public interest litigation, which greatly minimizes the complexity of handling environmental cases. 

### 4.3. Constructing a Risk Communication Mechanism among Multiple Subjects Outside the Litigation Process

As mentioned above, China has pioneered the Three Simultaneities system, which emphasizes minimizing the magnitude and likelihood of the damage of risks before they occur. In addition to the vertical domination of power, a horizontal communication mechanism between equal subjects should be established. Even if the public power sector is involved in this communication mechanism, it appears as an interlocutor, like a civil subject. The 2014 revision of China’s Environmental Protection Law provides for “Information Disclosure and Public Participation.” The official channels for public participation are illustrated in the Measures for Public Participation in Environmental Protection, released in 2015 and the Measures for Public Participation in Environmental Impact Assessment, released in 2018.

In China’s current practice, a variety of systems have been put in place to protect public participation, such as consultation, questionnaires, expert discussions, and hearings. However, these forms are often ineffective for environmental protection, which involves a wide range of issues and has far-reaching effects. Due to the failure of these forms, several mass movements involving environmental protection have emerged in China in recent years, such as the protest against the PX project in Xiamen in 2007, the protest against the waste incineration plant in Panyu District, Guangzhou in 2009, and the protest against the PX project in Dalian in 2011 [36]. These well-publicized environmental mass events highlight the public’s distrust of scientific knowledge, especially questioning the authority of experts and technocrats. Risk is a socially constructed product, not something that originally existed as an entity in nature. For example, a specific person’s responsibility for spreading environmental pollution is a result of responsibility for “other people” (rather than the natural environment).

Under this premise, it is necessary to introduce the mechanism of risk communication in the future environmental rights remedy in advance and to seek the appropriate solution in consideration of economic, environmental and value factors. Such a communication mechanism should not deprive stakeholders of the right to participate and be informed on the grounds that it requires more specialized knowledge. In the mechanism of equal communication, it is proposed to make a new positioning of the role of the public. In other words, under the uncertainty of scientific knowledge, the participating public should be transformed from a “receiver” of scientific knowledge to a “chooser” based on rational reflection, and the one-way information transmission model should be transformed into an interactive and cooperative model, addressing the cognitive value of the general public in environmental risk issues [37]. It should be underlined that this way of thinking is well grounded in the fields of risk psychology and risk sociology. In risk theories, risk perception and acceptance are two interconnected links, emphasizing that risk is not a purely objective matter but emerges based on public perceptions and reactions [38]. Then, risk communication as a means of addressing risks is an essential step beyond technical ones, and China is bound to change in this direction.

A wide range of social actors bears the consequences of environmental risks, and their damage to the natural environment may even involve intergenerational equity, affecting future generations of human beings. Therefore, the unequal distribution of responsibilities requires an appropriate institutional design in response, which requires effective communication and prior participation. Theoretically, it has been argued that the reflexive communication mechanism should be introduced into environmental risk management to find a balance between the expert model and the public model and consider the interface between the social and state levels [39]. Thus, we can see a complementary triangle involving the courts, administrative agencies, and the proper subject of public interest litigation, which is usually a public interest organization or an individual in a specific case. The operation of this prior communication mechanism secures the possibility of balancing interests outside of public interest litigation for civil and administrative environmental cases. When it comes to making decisions about complex problems in the natural and social environment, the political and legal systems should be the first to take responsibility, which requires the ability to attribute and control risks, while the public is incompetent in this regard. However, this incompetence does not produce a black box, the public’s right to know is still reserved, and the monopoly of knowledge cannot be used to exclude the possibility of citizens’ supervision. The communication mechanism is to introduce risk communication procedures in the institutional operation of the rights remedy, which can effectively avoid the marginalization of public participation.

Mass incidents related to environmental issues occur because the public, enterprises, government and experts differ in their perceptions of environmental risks. This cognitive dissonance leads to difficulties connecting actions and even conflicts between them. Risk communication comprises three dimensions: communication between government departments and the public, communication between social groups with the involvement of experts, and communication between judicial departments and administrative departments. It can resolve differences through effective communication between enterprises, government, experts and the general public, and it can eliminate disparities in risk perceptions outside the litigation process. In terms of effectiveness, it can prevent and respond to risks and eliminate mass incidents at the root more efficiently than using power.

## 5. Conclusions

Environmental protection and the maintenance of biodiversity are major issues worldwide. Countries have adopted legislative, administrative and judicial measures to achieve this goal as an important social mechanism. As this paper examines the practical experience of other countries reveals, judicial power and administrative power share the same legislative basis, and they are not in an either–or relationship, but in a collaborative relationship with a division of labor. These two types of public power are likely to converge in environmental tort litigation. According to the latest statistical report for 2021, the number of first-instance cases accepted in China’s environmental civil, environmental administrative, environmental criminal, environmental public interest litigation, and ecological and environmental damage compensation cases increased by 10.18%, 23.42%, -4.33%, 25.97%, and 131.50% [40].

By summarizing China’s environmental rights remedy system and comparing the experiences of other countries, this paper attempted to draw a clear line between judicial power and administrative power. Environmental tort liability in modern society is significantly different from conventional civil, criminal, and administrative liabilities. The remedy of environmental rights requires efficient initiatives and a wealth of knowledge and experience, which environmental administrations are most familiar with. In this regard, we should fully respect administrative power and set up a review process before litigation.

In contrast, environmental justice should adhere to its boundaries and serve as a neutral adjudicator for specific cases and interests rather than delegating too much responsibility or directly replacing administrative power. In addition, it is also necessary to consider the position of experts in environmental litigation and risk communication procedures outside of litigation to provide more abundant institutional guarantees for the remedy of environmental rights.

## Data Availability

Not applicable.
